# Digital Self-Monitoring Tools for the Management of Gestational Weight Gain: Protocol for a Systematic Review

**DOI:** 10.2196/50145

**Published:** 2023-10-26

**Authors:** Jan Mooney, Alicia A Dahl

**Affiliations:** 1 Department of Psychological Science College of Liberal Arts and Sciences University of North Carolina at Charlotte Charlotte, NC United States; 2 Department of Public Health Sciences College of Health and Human Services University of North Carolina at Charlotte Charlotte, NC United States

**Keywords:** protocol, systematic review, gestational weight gain, self-monitoring, digital health, review methods, review methodology, pregnant, pregnancy, gestational, weight, maternal, mobile phone

## Abstract

**Background:**

Gestational weight gain (GWG) exceeding the recommendations of the Institute of Medicine (in the United States) is associated with numerous adverse maternal and infant health outcomes. While many behavioral interventions targeting nutrition and physical activity have been developed to promote GWG within the Institute of Medicine guidelines, engagement and results are variable. Technology-mediated interventions can potentially increase the feasibility, acceptability, and reach of interventions, particularly for pregnant women, for whom integration of interventions into daily life may be critical to retention and adherence. Previous reviews highlight GWG self-monitoring as a common intervention component, and emerging work has begun to integrate digital self-monitoring into technology-mediated interventions. With rapid advances in technology-mediated interventions, a focused synthesis of literature examining the role of digital self-monitoring tools in managing GWG is warranted to guide clinical practice and inform future studies.

**Objective:**

The proposed review aims to synthesize the emerging research base evaluating digital GWG self-monitoring interventions, primarily focusing on whether the intervention is effective in managing GWG. Depending on the characteristics of the included research, secondary focus areas will comprise intervention recruitment and retention, feasibility, acceptability, and differences between stand-alone and multicomponent interventions.

**Methods:**

This protocol was developed following the PRISMA-P (Preferred Reporting Items for Systematic Review and Meta-Analysis Protocols) guidelines for systematic review protocols. The proposed review would use a planned and systematic approach to identify, evaluate, and synthesize relevant and recent empirical quantitative studies (reported in English) examining the use of digital weight self-monitoring tools in the context of technology-mediated interventions to manage GWG in pregnant US adults, with at least 2 instances of data collection. Literature eligible for inclusion will have a publication date between January 2010 and July 2020. The Effective Public Health Practice Project Quality Assessment Tool for Quantitative Studies will be used to assess the methodological quality of included studies across various domains, and results will be synthesized and summarized per the synthesis without meta-analysis guidelines.

**Results:**

The initial queries of 1150 records have been executed and papers have been screened for inclusion. Data extractions are expected to be finished by December 2023. Results are expected in 2024. The systematic review that will be generated from this protocol will offer evidence for the use of digital self-monitoring tools in the management of GWG.

**Conclusions:**

The planned, focused synthesis of relevant literature has the potential to inform the use of digital weight self-monitoring tools in the context of future technology-mediated interventions to manage GWG. In addition, the planned review has the potential to contribute as part of a broader movement in research toward empirically supporting the inclusion of specific components within more extensive, multicomponent interventions to balance parsimony and effectiveness.

**Trial Registration:**

PROSPERO CRD42020204820; https://tinyurl.com/ybzt6bvr

**International Registered Report Identifier (IRRID):**

PRR1-10.2196/50145

## Introduction

### Background

Gestational weight gain (GWG) is currently assessed by comparison to recommendations provided by the Institute of Medicine (IOM) [1]. These guidelines are based on prepregnancy BMI, such that GWG falling below the recommended range is considered “inadequate,” and GWG above the recommended range is considered “excessive.” Excessive gestational weight gain has demonstrable associations with adverse maternal and infant health outcomes, such as pregnancy complications (eg, hypertension and preeclampsia) [2-4], postpartum weight retention with implications for long-term maternal obesity [5], large-for-gestational-age [2,3,6], macrosomia [6], cesarean delivery [2,3,6], elevated child BMI [5,7,8], among others.

Recent systematic reviews have primarily supported health behavior change (eg, diet or physical activity) to reduce the prevalence of excessive GWG [9-13]. However, these multiple review papers have described concerns about the generalizability of findings due to the inclusion of studies characterized by small sample sizes, low participant recruitment and retention, lack of diversity, or a lack of information about these areas [10,11,14-17]. Moreover, the prevalence of excessive gestational weight gain remains high, with nearly half (48%) of pregnant women in the United States reporting weight gain above the recommended ranges across categorizations of prepregnancy BMI [18-20]. A recent meta-analysis of GWG-focused interventions during pregnancy [21] reflected a notably nonrepresentative sample (over 80% White women). Homogenous samples such as these call for researchers to assess how specific interventions have addressed acceptability, feasibility, and effectiveness for certain subpopulations. Notable efforts to recruit and test different lifestyle intervention strategies to modify GWG in diverse populations have been successful through a consortium model [22].

As new technologies emerge, technology-mediated interventions have been proposed as a potential solution for expanding the reach and scope of behavioral and interpersonal (eg, provider-directed) interventions [12,23-25]. Internet websites, text messaging, and smartphone apps have been identified as accessible resources for a diverse population of racial or ethnic and socioeconomic groups [24,26], and other potential methods may include activity and lifestyle tracking technologies such as wearable devices or scales with Bluetooth or other wireless capabilities. For example, a technology-based intervention that incorporated skills training, prompts, personalized feedback, counseling, and peer support via text messaging, health coaching phone calls, and Facebook group involvement was noted to be a significant improvement over usual care concerning managing weight during pregnancy for urban mothers with low-income [27,28]. Such findings underscore the importance of health education and awareness around GWG outcomes for low-income women [29] and the need for complex intervention development that is adequately informed by and responsive to the risks and needs of the target population [30].

Recent reviews have specifically examined the impact of technology-supported lifestyle interventions on GWG and postpartum weight retention [31-34]. Lau et al [31] determined e-based lifestyle interventions to be acceptable among pregnant populations, calling for future work to optimize intervention components. Mertens et al [32] focused mainly on randomized controlled trials (RCTs) using telecoaching or feedback (eg, via SMS or app) that was responsive to participants’ self-monitoring, with some evidence of optimization for GWG. In their review of exclusively digital health interventions (for managing GWG, improving diet, or increasing physical activity), Rhodes et al [33] highlighted that while self-monitoring components were not universally included among the effective interventions, 7 out of 11 interventions did use some self-monitoring component, revealing a potential avenue for a more nuanced understanding of the impact of specific mobile health (mHealth) approaches in managing GWG. The results indicate that in the context of marked heterogeneity in outcomes studied, digital interventions did not demonstrate evidence of effectiveness for managing GWG, highlighting that different types (ie, subareas) of digital approaches may show differing levels of effectiveness [33]. Furthermore, findings of a meta-analysis determined e-Health technology exposure was associated with a nonsignificant effect on weight management during pregnancy but in postpartum, there was a statistically significant weight reduction [34].

Technological or digital interventions may provide feasible solutions for adapting to unique life circumstances and needs [35]. However, a more focused assessment of the degree to which specific technological approaches are feasible, acceptable, and effective are essential prerequisites [36]. Despite the generally broad availability and accessibility of mobile phone technology among women in the United States [37], there are significant considerations for addressing participation barriers associated with technology, such as rural versus nonrural users, inconsistent data plans, and device sharing [38,39]. Consequently, specific characteristics and components of interventions (eg, social media versus smartphone apps or responsive wearable devices) may dictate accessibility and constrain the reach of digital approaches [40,41].

Given the high overall rate of mobile phone ownership and use and qualitative data suggesting that pregnant women desire personalized mobile tools for healthy lifestyle behaviors in pregnancy [42], mHealth interventions represent a unique subclass of interventions distinct from other technology-based interventions [43]. Consistent with this sentiment, the World Health Organization, in their guideline on digital interventions to strengthen health systems, specifically addressed “targeted client communication for behavior change” as a context-aware recommendation for the use and development of mHealth technologies (eg, text messaging, interactive content such as apps, games, and social media, wireless devices) [44]. Pregnancy-related smartphone apps are the largest category of medically related apps, and smartphone apps with interactive capability are the most popular and well-liked [45]. While many smartphone apps advertise themselves as focused on GWG self-monitoring, a recent review highlighted that only a small percentage provided all of the key features of GWG monitoring (ie, tracking tools and guidelines or recommendations) [46].

A systematic review examining weight self-monitoring in nonpregnant populations seeking weight loss or management noted that the use of weight self-monitoring differs considerably between studies concerning aspects such as frequency, tools used, instructions provided, and role of weight self-monitoring (ie, as the only intervention versus as one part of a multicomponent intervention) [47]. Overall, across studies, participants perceived weight monitoring positively and feasibly. However, Zheng et al [47] highlighted the variability and inaccuracies that may present with self-report assessments of weighing behavior and weight and joined a broader call for more objective assessment methods. Moreover, the use of “smart” (ie, cellular-connected) scales tied to personalized feedback has been associated with weight loss, adherence to weight self-monitoring, and decreases in caloric consumption in comparison to a waitlist control group [48]. Thus, digital health approaches to weight self-monitoring have the potential to address the need for objectivity and improve adherence while maintaining the feasibility associated with self-monitoring.

Notably, the goals of pregnant individuals engaged in a GWG management program may differ considerably to those seeking weight loss or management outside of pregnancy. This highlights the need to closely examine and understand the impact of weight self-monitoring in this unique population. While a systematic review has been conducted to investigate the effect of routine weighing during pregnancy as a standalone intervention to manage GWG, this review (which included only 2 published RCTs) included both self-weighing and clinician weighing, was unable to identify any studies using digital health approaches to weight self-monitoring, and ultimately failed to support the effectiveness of weight monitoring for GWG management [49]. In a subsequent comment, the authors acknowledged that while the systematic review results were inconclusive, research in this area continues to accumulate [50,51]. In sum, though digital approaches provide a method to maintain benefits previously observed across pregnant and nonpregnant populations (about weight monitoring) while also increasing feasibility and adherence, uncertain quality of consumer-facing options (ie, publicly available smartphone apps) and a rapidly evolving research base highlight the need to evaluate the potential role of digital self-monitoring tools for the management of GWG.

Thus, the proposed review aims to summarize and synthesize the emerging research base evaluating digital GWG self-monitoring interventions for managing GWG during pregnancy. The primary outcome focus of this systematic review is GWG because prior RCT methods targeting nutrition or physical activity have yielded promising but modest effects on the reduction of GWG by about 20% [11]. There is a need to identify more effective and sustainable approaches to intervention delivery.

### Objectives

#### Overview

The proposed review would aim to answer the following research questions relating to interventions, including a digital GWG self-monitoring component with a primary or secondary focus on the management of adult GWG.

#### Primary Focus

- How effective are interventions, including digital self-monitoring tools, in the management of GWG?

#### Secondary Foci

- Are interventions including digital GWG self-monitoring components associated with increased retention of participants in comparison to usual care or other care in control groups?
- Are interventions including digital GWG self-monitoring components associated with indicators of feasibility and acceptability among participants?
- Are there differences in the effects of standalone digital GWG self-monitoring interventions compared to multicomponent interventions, including digital GWG self-monitoring components?
- How diverse are the samples used to study GWG self-monitoring with digital components?

## Methods

### Study Designs

This protocol followed the PRISMA-P (Preferred Reporting Items for Systematic Review and Meta-Analysis Protocols) guidelines for developing systematic review protocols (the PRISMA-P checklist is provided herewith). Studies will be selected according to the following criteria. The proposed review would include RCTs, feasibility trials, acceptability trials, pilot studies, nonrandomized trials, and controlled before-and-after studies. The proposed review will consist of studies examining adult pregnant individuals (18 years or older, as adolescent growth and development may further complicate the measurement of GWG and associated recommendations). It will include studies that examine individuals with chronic conditions, diseases, or disorders, as long as the primary or secondary outcome relates to GWG. Studies will be selected for inclusion in the proposed review only if at least 2 time points of data are available (ie, no studies that report only on cross-sectional data would be included). Additionally, though no restrictions will be placed on the length of follow-up for outcomes, follow-up data will be reported if available.

### Interventions

Of interest in the proposed review would be mHealth interventions for the management of GWG, which include a digital weight self-monitoring component (eg, mobile app weight tracker). For the proposed review, interventions will be considered “mHealth” if the primary components of the intervention is delivered via a wireless device (eg, tablet, iPad, wearable device, and wireless scale) or mobile telephone (eg, cell phone, smartphone, and mobile website), and would be considered to have a weight self-monitoring component if they require the user to enter their weight, whether these data are collected automatically or input manually by the user. However, interventions that do not require any interactional component (ie, do not use the advanced functionality in mobile or wireless devices, eg, appointment reminders and static informational websites accessed via mobile devices) would not be considered mHealth interventions. Given the proposed review’s focus on a descriptive (rather than reductive) summary of the extant literature on the use of digital self-monitoring technologies in interventions for the management of GWG, studies with any comparison group are eligible for inclusion, as are studies with no comparison group.

### Evaluation Outcomes

In the proposed review, included studies would have an outcome relating to GWG as the primary study outcome (ie, interventions that attempt to prevent excessive GWG or promote adequate GWG within IOM guidelines). For the proposed review, GWG would be considered to be reflected by the total weight gain from pre- or early pregnancy to the time of delivery, the rate of weight gain per week from pre- or early pregnancy to the time of delivery, or the IOM categorization of the adequacy of pregnancy weight gain. Secondary study outcomes (if provided) would include physical activity or exercise behaviors (measured indirectly via self-reported or directly via activity tracker data reflecting frequency, intensity, or duration), diet or eating behaviors or diet quality (measured via self-reported dietary intake records or food frequency questionnaires, reflected as nutrient or energy intakes or as evaluation of dietary quality in comparison to established guidelines, eg, Healthy Eating Index-2015), sleep behaviors (measured indirectly via self-reported or directly via activity tracker data reflecting duration or quality), or psychosocial well-being or mood (measured via self-report surveys).

### Exclusion Criteria

The proposed review will be restricted to studies conducted within the United States, given how guidelines for GWG vary considerably between countries [52]. We will include papers reported in English only, though a list of possibly relevant titles in other languages will be provided as a supplemental resource. Inclusion and exclusion criteria are summarized in Textbox 1.

**Inclusion or exclusion criteria.**

**Included criteria**

- **Paper type:** Randomized controlled trials, feasibility trials, acceptability trials, pilot studies, nonrandomized trials, and controlled before-and-after studies
- **Sample:** Adult pregnant individuals 18 years or older, with or without chronic health conditions
- **Outcomes:** Primary or secondary outcomes relate to gestational weight gain
- Additional psychosocial or behavioral outcomes, including physical activity or exercise, diet or eating, sleep, or well-being or mood
- **Study timespan:** Longitudinal or prepost designs, with or without postpartum follow-up period
- **Intervention:** mHealth interventions focused on managing gestational weight gain; primary components of the intervention are delivered over the wireless device or mobile telephone; participants are required to monitor weight (either automatically or via manual entry)
- **Comparison:** Studies with and without intervention comparison groups (eg, waitlist control group, treatment-as-usual, no comparison group)
- **Study location:** Conducted in the United States
- **Language:** English

**Excluded criteria**

- **Paper type:** Qualitative studies, studies that do not contain an intervention component
- **Sample:** Adolescent pregnant individuals, nonpregnant individuals
- **Outcomes:** No outcomes relating to gestational weight gain or gestational weight gain was not measured
- **Additional outcomes:** Not applicable, if other outcomes are collected, they will not be reported (eg, blood pressure and blood glucose)
- **Study timespan:** Cross-sectional studies
- **Intervention:** No digital weight self-monitoring component; no interactional component; static informational websites
- **Comparison:** Not applicable
- **Study location:** Conducted outside of the United States
- **Language:** Languages other than English (list of possibly relevant titles published in other languages will be included as a supplemental resource)

### Search Strategy

Electronic databases will be searched for literature published between January 2010 and July 2020 (to encompass the past decade given the rapid and ongoing development of digital health technology): PubMed or MEDLINE, Web of Science, Google Scholar, CINAHL, Academic Search Complete, PsycINFO, Embase. Both keywords and MeSH (Medical Subject Headings) terms relating to GWG, eHealth, mHealth, and variations of these terms. Boolean logic (ie, use of AND Mesh OR) will be implemented where appropriate.

Reference lists of studies initially selected for the review and lists of studies citing the studies originally chosen for the review will be assessed to identify additional studies that may meet eligibility criteria. Additionally, studies included in extant systematic reviews relevant to the proposed review will be evaluated against the proposed eligibility criteria. Finally, authors of registered protocols (for which published works summarizing results cannot be located) will be contacted to solicit any unpublished or unfinished work that may be incorporated into the narrative synthesis.

A university librarian with content coverage in public health provided preliminary consultation in developing the search strategy to ensure it captured all relevant terms. An example of the PubMed database search strategy is provided in Multimedia Appendix 1. It will be amended for other search engines to use database-specific or -compliant subject headings and keywords (ie, controlled vocabularies).

### Data Management

Excel (Microsoft Corp) will be used to review literature search result lists. These results from all searched databases will be combined, and duplicate entries will be removed. The process will be described using a PRISMA (Preferred Reporting Items for Systematic Review and Meta-Analysis) flow diagram. A checklist based on the inclusion and exclusion criteria will be created to facilitate the initial title screening of papers. Further, 1 reviewer will independently screen titles. In total, 2 reviewers will screen the abstracts associated with the resultant list of relevant titles to identify studies for which the entire text will be reviewed. The Kappa statistic will be used to assess the inter-reviewer agreement on this list before pulling the selected studies' full text. Finally, studies for which full text is reviewed will be independently assessed for inclusion by 2 reviewers, with any discrepancies brought to a third reviewer for additional feedback to reach a consensus on the final list of included studies.

### Quality Assessment

The Effective Public Health Practice Project Quality Assessment Tool for Quantitative Studies will be used to assess the methodological quality (indicated as “strong,” “moderate,” or “weak”) of selected studies in each of the following areas: study design, analysis, withdrawals and dropouts, data collection practices, selection bias, invention integrity, blinding as part of a controlled trial, and confounders. Both reviewers will conduct quality assessments, and any discrepancies will be brought to a third reviewer for additional feedback to reach a consensus on quality evaluation. Additionally, these ratings will be presented in a tabular format to aid in contextualizing the narrative summary. Ratings over 2 SDs below the mean quality assessment rating will be excluded from any synthesis of outcome data.

### Data Extraction and Synthesis

Descriptive details of interventions, including details of this study (setting and length), participant characteristics, the overall role of digital self-monitoring technology in the intervention, features of technology used in the intervention, description of any other intervention components, and outcomes will be extracted for narrative synthesis. Further, 2 reviewers will independently extract relevant information. Disagreements on the categorizations for selected studies will be discussed, and a third reviewer will be consulted as necessary until a consensus is reached. See Multimedia Appendices 2 and 3 for the data extraction tool and variables of interest.

A summary of findings and evaluation of the strength of the body of evidence will be provided in accordance with the synthesis without meta-analysis guidelines [53] in narrative and tabular format based on the outcomes reported in the studies reviewed, along with an indication of the quality of the studies as indicated by the Effective Public Health Practice Project Quality Assessment Tool. For continuous outcomes (eg, total GWG, rate of GWG per week), means and SDs would be used for comparison purposes. For categorical outcomes (eg, adequacy of GWG according to IOM guidelines), relative risk or odds ratios would be used for comparison purposes. If at least 2 studies are available examining intervention impact on pregnant women categorized as “overweight” or “obese” based on currently used BMI categorizations, a separate narrative synthesis section would evaluate intervention use and outcomes in this subpopulation.

## Results

The research protocol was registered with PROSPERO (CRD42020204820), on September 29, 2020. The initial queries resulting in 1150 total records have been screened. Data extractions are expected to be finished by December 2023. The systematic review generated from this protocol will offer evidence for using digital self-monitoring tools in the management of GWG. The research to practice translation may have implications for OB or GYN care by summarizing the feasibility and acceptability of digital self-monitoring tools for GWG. The results will be disseminated through a peer-reviewed publication, expected in 2024.

## Discussion

### Principal Results

While advancements in intervention science have primarily supported the use of behavior change techniques (eg, physical activity and diet changes) for the management of GWG from the perspective of efficacy, much uncertainty remains regarding their broader feasibility and acceptability, as well as their impact on related health outcomes (eg, pregnancy and birth complications), constraining evidence for more general effectiveness. However, the continuing and rapid development of new intervention approaches, particularly in the area of weight self-monitoring mHealth technology (given its accumulated support in nonpregnant populations), necessitate an updated and focused systematic review to more specifically address the potential of digital weight self-monitoring technology to address current gaps in feasibility, acceptability, and broad reach of interventions for managing GWG. If the proposed review finds that digital weight self-monitoring components are associated with desirable GWG outcomes or with improvements or maintenance of health behaviors (secondary outcomes), this would suggest that digital weight self-monitoring is an essential and necessary component of technology-mediated interventions focused on GWG.

### Comparison With Prior Work

While previous reviews have examined effectiveness in the broadly defined use of e- and mHealth technologies to intervene concerning a variety of pregnancy health outcomes, limited consideration of such aspects as acceptability, feasibility (eg, retention), participant perceptions, and unique features specific to digital self-monitoring interventions uniquely positions the proposed review as both an extension of prior work and a much-needed reorientation toward a more practical understanding of the utility of digital self-monitoring tools for GWG management.

### Limitations

The proposed review would include non-RCT studies, which may reduce the number of included studies with a comparison group and limit the conclusions that can be drawn regarding the impact of digital GWG self-monitoring interventions. Further, much of the research conducted to date (as suggested by previous reviews) has occurred with mostly White pregnant individuals. Consequently, any systematic reviews focusing on this body of literature will likely have limited applicability for Black, indigenous, or other racial or ethnic minority groups [54]. Given the notion of the “digital divide,” it is possible that larger socioeconomic systems may dictate access to specific technologies.

Similarly, digital weight self-monitoring tools are primarily designed from a personal behavior change perspective. Thus, research captured in the context of the proposed review may not reflect how sociocultural systems (eg, familial or cultural context, interpersonal relational dynamics) may shape individuals’ perceptions of and engagement with the apps. Finally, the authors acknowledge that their socioeconomic positions, racial or ethnic identities, and life histories may constrain the degree to which they can recognize, acknowledge, and consider the unique contexts and barriers experienced by socioeconomically, racially, ethnically, and culturally diverse pregnant women who do and do not engage with pregnancy weight management interventions.

### Conclusions

The proposed review could help identify the degree to which digital GWG self-monitoring (as reflected by published empirical studies) can impact the prevalence of healthy GWG and the degree to which participants find the interventions feasible and acceptable. Furthermore, identifying effective strategies within interventions can inform future intervention research and, in the long term, individualized selection of efficacious interventions (and their constituent components) in clinical practice settings.

## Appendix

Multimedia Appendix 1 PubMed search strategy.

Multimedia Appendix 2 Data extraction tool—study details.

Multimedia Appendix 3 Data extraction tool—study outcomes.

Multimedia Appendix 4 PRISMA-P Checklist.

## Abbreviations

GWG: gestational weight gain
IOM: Institute of Medicine
MeSH: Medical Subject Headings
mHealth: mobile health
PRISMA: Preferred Reporting Items for Systematic Reviews and Meta-Analyses
PRISMA-P: Preferred Reporting Items for Systematic Reviews and Meta-Analyses Protocols
RCT: randomized controlled trial

## Data Availability

Data sharing does not apply to the above-described protocol, as no data sets were generated or analyzed in the production of the protocol. The proposed data set described in the protocol would consist of information gathered primarily from published research. Any additional data generated during the proposed systematic review (eg, quality ratings for specific papers) will be described as appropriate within the paper describing the review results. Any data not related will be available from the corresponding author upon reasonable request.
