# Supplementary material for: Digital Self-Monitoring Tools for the Management of Gestational Weight Gain: Protocol for a Systematic Review
Source: JMIR Res Protoc. 2023 Oct 26;12:e50145. doi: 10.2196/50145 (PMC10636618; doi:10.2196/50145)
Supplement: Multimedia Appendix 1 [file resprot_v12i1e50145_app1.docx]

**Multimedia Appendix 1**. PubMed search strategy.

Query:

(((("mobile app*"[All Fields]) OR ("Telemedicine"[MeSH Terms]) OR ("Mobile applications"[MeSH Terms]) OR ("mobile device"[All Fields]) OR ("Cell Phone"[MeSH Terms]) OR (“digital”[All Fields]) OR (“technology”[All Fields]) OR ("wearable*"[All Fields])) AND (("tracking"[Other Term]) OR ("self-monitoring"[All Fields]) OR (“monitoring”[All Fields]) OR “Self-Management”[MeSH Terms]) OR (“weight tracker”[All Fields]) AND (("gestational weight gain"[All Fields]) OR ((("pregnancy"[All Fields]) OR ("Pregnancy"[MeSH Terms])) AND ("weight gain"[All Fields])) OR ("Maternal Health"[MeSH Terms]))) AND (("2010/1/1"[Date - Publication] : "2020/7/1"[Date - Publication]))) AND ("english"[Language])
