# Supplementary material for: Digital Self-Monitoring Tools for the Management of Gestational Weight Gain: Protocol for a Systematic Review
Source: JMIR Res Protoc. 2023 Oct 26;12:e50145. doi: 10.2196/50145 (PMC10636618; doi:10.2196/50145)
Supplement: Multimedia Appendix 2 [file resprot_v12i1e50145_app2.docx]

**Multimedia Appendix 2.** Data extraction tool—study details.

| **Title** | **Geo Region** | **Design** | **Target Pop** | **Targets of Intervention** | **Inclusion Criteria** | **Exclusion Criteria** | **Method/Details of Digital Weight Self-Monitoring** | **Intervention Components** | **Control Group Activities** | **Size of Interv. Group** | **Size of Control Group** | **Participant Demographics (age, race/eth., education, income)** | **Number Multiparous Women** | **Gestational Week of Intervention Start** |
| --- | --- | --- | --- | --- | --- | --- | --- | --- | --- | --- | --- | --- | --- | --- |
|  |  |  |  |  |  |  |  |  |  |  |  |  |  |  |
