# Supplementary material for: Digital Self-Monitoring Tools for the Management of Gestational Weight Gain: Protocol for a Systematic Review
Source: JMIR Res Protoc. 2023 Oct 26;12:e50145. doi: 10.2196/50145 (PMC10636618; doi:10.2196/50145)
Supplement: Multimedia Appendix 3 [file resprot_v12i1e50145_app3.docx]

**Multimedia Appendix 3.** Data extraction tool—study outcomes.

| **GWG Intervention Length**  **(e.g., 10 weekly Lessons)** | **Data Collection Timepoints** | **How Often Weight Monitor** | **Attrition Rate** | **Engagement Measured** | **Primary Outcome and Measure** | **Secondary Outcome and Measure** | **Intervention Vs. Control Weight Difference** | **Interpretation Of Weight Difference** |
| --- | --- | --- | --- | --- | --- | --- | --- | --- |
|  |  |  |  |  |  |  |  |  |
